# Transcriptome-Based Weighted Gene Co-Expression Network Analysis Reveals the Photosynthesis Pathway and Hub Genes Involved in Promoting Tiller Growth under Repeated Drought–Rewatering Cycles in Perennial Ryegrass

**DOI:** 10.3390/plants13060854

**Published:** 2024-03-15

**Authors:** Yunjia Ding, Xiaxiang Zhang, Jialei Li, Ruying Wang, Jie Chen, Lingna Kong, Xin Li, Zhimin Yang, Lili Zhuang

**Affiliations:** 1 College of Agro-Grassland Science, Nanjing Agricultural University, Nanjing 210095, China; 2018120002@njau.edu.cn (Y.D.);; 2 Department of Horticulture, Oregon State University, Corvallis, OR 97331, USA; 3 College of Horticulture, Nanjing Agricultural University, Nanjing 210095, China; 4 National Experimental Teaching Center for Plant Production, Nanjing Agricultural University, Nanjing 210095, China; 5 College of Life Science, Nanjing Agricultural University, Nanjing 210095, China

**Keywords:** perennial ryegrass, drought stress, rewatering, tiller development, transcriptome, weighted gene co-expression network analysis (WGCNA)

## Abstract

Drought stress, which often occurs repeatedly across the world, can cause multiple and long-term effects on plant growth. However, the repeated drought–rewatering effects on plant growth remain uncertain. This study was conducted to determine the effects of drought–rewatering cycles on aboveground growth and explore the underlying mechanisms. Perennial ryegrass plants were subjected to three watering regimes: well-watered control (W), two cycles of drought–rewatering (D2R), and one cycle of drought–rewatering (D1R). The results indicated that the D2R treatment increased the tiller number by 40.9% and accumulated 28.3% more aboveground biomass compared with W; whereas the D1R treatment reduced the tiller number by 23.9% and biomass by 42.2% compared to the W treatment. A time-course transcriptome analysis was performed using crown tissues obtained from plants under D2R and W treatments at 14, 17, 30, and 33 days (d). A total number of 2272 differentially expressed genes (DEGs) were identified. In addition, an in-depth weighted gene co-expression network analysis (WGCNA) was carried out to investigate the relationship between RNA-seq data and tiller number. The results indicated that DEGs were enriched in photosynthesis-related pathways and were further supported by chlorophyll content measurements. Moreover, tiller-development-related hub genes were identified in the D2R treatment, including *F-box/LRR-repeat MAX2* homolog (*D3*), *homeobox-leucine zipper protein HOX12-like* (*HOX12*), and *putative laccase-17* (*LAC17*). The consistency of RNA-seq and qRT-PCR data were validated by high Pearson’s correlation coefficients ranging from 0.899 to 0.998. This study can provide a new irrigation management strategy that might increase plant biomass with less water consumption. In addition, candidate photosynthesis and hub genes in regulating tiller growth may provide new insights for drought-resistant breeding.

## 1. Introduction

The shortage of freshwater resources has become a prominent problem affecting agricultural and industrial production. Drought stress significantly limits plant growth and productivity. Yield losses caused by drought stress in the field typically range between 30% and 90% [1]. Plants have developed various strategies to adapt to drought stress. Plants detect environmental signals through a variety of mechanisms and activate responses accordingly at the molecular, physiological, and biochemical levels to withstand and adapt to drought [2]. Drought stress adversely affects many vital plant developmental processes, including leaf initiation, plant height elongation, and tiller development [3,4].

Severe and prolonged drought is detrimental to plants; however, plants usually encounter cycles of mild drought events throughout the growing season and recover when the water supply is replenished [5,6]. Interestingly, research has shown that recurrent drought stress can improve the overall drought tolerance of plants. Plants can form a physiological, transcriptomic, proteomic, metabolomic, lipidomic, or epigenetic ‘stress memory’ during a pre-stress event, which may allow for a more rapid response when the stress recurs, such as in *Arabidopsis thalian*, *Zea mays*, *Beta vulgaris*, *Triticum aestivum*, and *Polygonum persicaria* [7,8,9,10,11]. For example, plants subjected to repeated cycles of dehydration stress exhibited both transcriptional and physiological memory responses during subsequent dehydration stress, including a reduced water loss rate, compared to plants experiencing dehydration stress for the first time in *Arabidopsis* and maize [8,12]. In other aspects, metabolites such as proline and sugars were highly accumulated under repeated drought stress [13]; changes in abscisic acid (ABA) and jasmonic acid (JA), as well as photosynthetic recovery, were observed in plants that had encountered previous drought events [7,14,15,16].

However, the enhancement of drought stress resistance usually comes at the cost of a decrease in plant growth. For example, repeated drought–rewatering (mainly with high drought intensity) restricts plant growth, such as in tall fescue (*Festuca arundinacea*), perennial ryegrass (*Lolium perenne*) [17], *Saccharum* spp. [18], and *Sorghum bicolor* [19]. In other cases, plants subjected to repeated drought–rewatering cycles may recover and outperform plants without drought stress. For example, the aboveground and belowground biomass of Phalaris (*Phalaris aquatica*) were comparable to the well-watered controls after recovery from repeated drought treatment [20]. In another study, greater or similar tiller numbers and biomass were observed from perennial grass (*Leymus chinensis*) subjected to repeated moderate drought and severe drought intensities compared to the well-watered control treatment [21]. Whether plant growth can partially recover, fully recover, or outperform depends on the intensity and duration of prior drought conditions; in addition, plant species, variety, and the number of consecutive drying cycles are also critical factors [21,22,23]. Thus, determining suitable drought–rewatering combinations that could promote plant growth with less water consumption is of great importance for agricultural productivity.

Perennial ryegrass is an important cool-season forage and turfgrass used worldwide. Drought is a major constraint to the growth and development of perennial ryegrass [24]. Tillering is an essential trait for crop yield, and it is also important for the rapid establishment and recovery of plants after they are subjected to stresses [25,26]. The development of tillers that initiate from crowns is controlled by endogenous factors and environmental factors [27,28]. The tiller growth of perennial ryegrass is sensitive to drought stress [29,30]. However, there were several studies that mentioned that tiller development could recover or increase in grasses under a repeated drought–rewatering treatment [20,21,31,32]. A perennial ryegrass accession from Norway had 50% more shoot dry matter than the next best-performing accession after six drought cycles [32]. We hypothesized that drought can negatively affect plant growth, but this may depend on the drought–rewatering cycles and drought severity. Thus, determining the conditions (drought duration, drought intensity, timing of rewatering, etc.) that could promote tiller growth are of significant importance. Additionally, hub genes and gene networks involved in tiller growth under repeated drought–rewatering cycles compared with consistent watering conditions have not been explicitly studied.

Therefore, this study aims to (1) evaluate the effects of well-watered control (W), two cycles of drought–rewatering (D2R), and one cycle of drought–rewatering (D1R) on perennial ryegrass tiller growth, as well as determine the suitable drought intensity for rewatering; (2) explore the underlying regulatory mechanism behind the enhanced tillering under D2R versus W treatment using a time-course comparative transcriptomics analysis at 14 (end of the first drought), 17 (end of 3 days after the first rewatering), 30 (end of the second drought), and 33 (3 days after the second rewatering) days (d). We demonstrate here that perennial ryegrass plants exposed to recurred drought events may perform better if watered at a suitable level of drought intensity. Meanwhile, hub genes and modules revealed by weighted gene co-expression network analysis (WGCNA) may serve as potential targets or markers for drought-resistant breeding under recurrent stress episodes.

## 2. Results

### 2.1. Effects of Drought–Rewatering Treatment on the Perennial Ryegrass Phenotype

**Figure 1:**
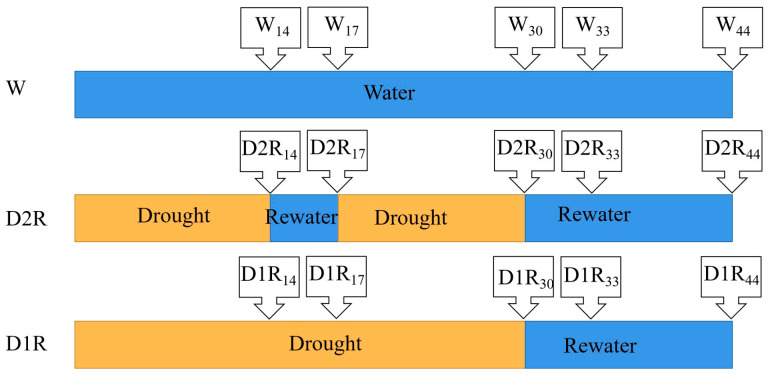
Diagram of well-watered control (W), two cycles of drought–rewatering (D2R), and one cycle of drought–rewatering (D1R) treatments. The boxes with arrows indicate each time point of the sampling and data collection, and the subscript number for each treatment denotes the sampling and data collection day within the 44 d experiment. W_14_, W_17_, W_30_, W_33_, and W_44_ indicates water control at 14, 17, 30, 33, and 44 d, respectively; D2R_14_: two cycles of drought–rewatering treatment at 14 d, which was at the end of the first drought stress; D2R_17_: two cycles of drought–rewatering treatment at 17 d, which was at the end of first 3 d rewatering; D2R_30_: two cycles of drought–rewatering at 30 d, which was at the end of the second drought stress; D2R_33_ and D2R_44_: two cycles of drought–rewatering treatment at 33 d and 44 d, which was indicated at 3 d and 14 d after the second rewatering, respectively; D1R_14_, D1R_17_, and D1R_30_ indicates 14, 17, and 30 d after drought stress, respectively; D1R_33_ and D1R_44_ indicates one cycle of drought–rewatering at 33 and 44 d, respectively, when plants were subjected to rewatering.

**Figure 2:**
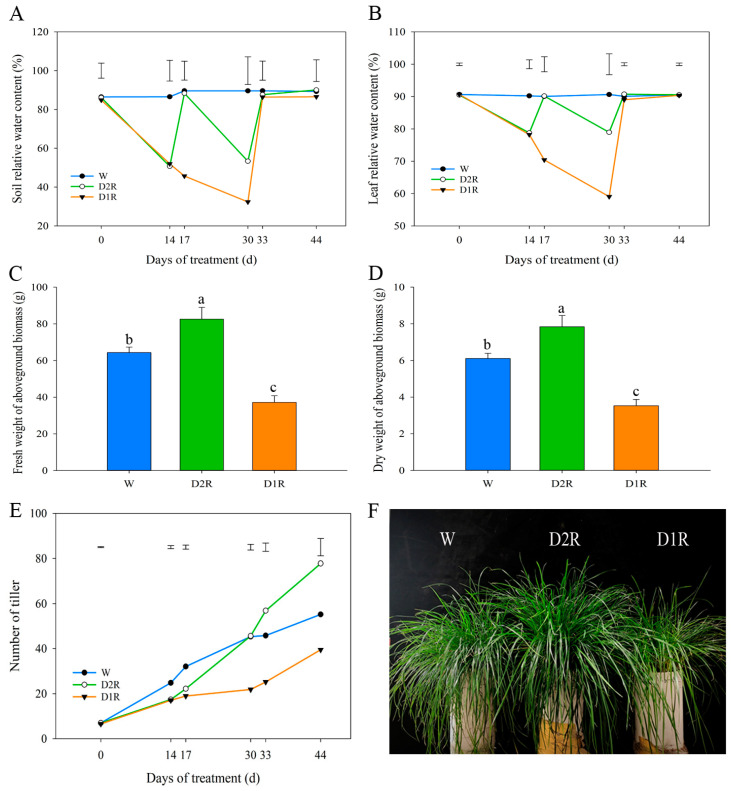
Agronomic traits of plants under well-watered control (W), two cycles of drought–rewatering (D2R), and one cycle of drought–rewatering (D1R) treatments. Soil relative water content was measured as gravimetric water content (**A**) and leaf relative water content (**B**) under W, D2R, and D1R treatments. Fresh weight (**C**) and dry weight (**D**) of aboveground biomass were harvested at 44 d of the experiment. (**E**) The number of tillers at each time point under W, D2R, and D1R treatments. (**F**) Aboveground growth comparison of perennial ryegrass under W, D2R, and D1R treatments at 44 d. Measurements were collected at 0, 14, 17, 30, 33, and 44 d of the experiment, and data collected at 14 and 30 d for D2R were at the end of the first and second drought stress, but before the first and second rewatering, respectively. Data represented the means across six replications. Vertical bars indicate LSD values (*p* < 0.05) for treatment comparisons on a given day of treatment in (**A**,**B**,**E**). In (**C**,**D**), vertical bars indicate the standard error of each treatment across six replications, and different lowercase letters above the columns indicate significant differences at *p* < 0.05.

To mimic natural drought and rewatering conditions, perennial ryegrass was exposed to different cycles of drought stress and subsequent recovery by rewatering (Figure 1). In the well-watered control (W), the soil relative water content (SRWC) was maintained at 86.5–89.4% (Figure 2A), and the leaf relative water content (LRWC) was maintained at about 90.0% (Figure 2B). In the D2R treatment, the SRWC decreased to 50.7% and 53.3% at D2R_14_ and D2R_30_, respectively. After rewatering, the SRWC increased to the control level at D2R_17_ and D2R_33_ (88.4% and 87.6%, respectively). Accordingly, the LRWC decreased to 78.8% and 78.9% at D2R_14_ and D2R_30_, respectively, and LRWC recovered to 90.2% and 90.7% at D2R_17_ and D2R_33_, respectively. Meanwhile, leaves exhibited a mild wilting symptom with D2R treatment at 14 and 30 d. In the D1R treatment, the SRWC gradually decreased to about 30.0% during the 30 d drought treatment and recovered to the control level after 3 d of rewatering (Figure 2A). The LRWC decreased from 90.7% to 59.1% when subjected to 30 d drought stress (D1R_30_), and LRWC increased to the same level as that of the control (about 90.0%) after rewatering at D1R_33_ (Figure 2B). Plants under D1R_30_ exhibited more severe drought stress, which was indicated by lower LRWC, compared to D2R_30_.

The fresh and dry weight (FW and DW, respectively) of aboveground biomass at 44 d of the experiment were measured. The FW and DW of the D2R treatment were significantly higher than those of the W control, whereas the FW and DW of the D1R treatment were significantly lower than those of the W control (Figure 2C,D). Tiller number per tube gradually increased from 7 to 55 during the 44 d period under W treatment (Figure 2E). Under D1R treatment, the tiller number increased relatively slowly and was significantly lower than that from W treatment after 30 d drought stress (22 tillers in D1R_30_ and 45 tillers in W_30_). After rewatering, the tiller number increased in the D1R treatment; however, only 42 tillers were found in D1R_44_ compared to 55 tillers in W_44_ (Figure 2E). In the D2R treatment, although the tiller number was significantly lower than that from W treatment after the first drought and rewatering cycle at D2R_14_ and D2R_17_, the growth rate of tillers increased remarkably compared with that in the W treatment after the second drought and rewatering cycle. The number of tillers in D2R increased by 24.1% and 40.9% compared with W at D2R_33_ and D2R_44_, respectively (Figure 2E). The phenotypic results indicated that plants subjected to D2R grew more vigorously compared to W and D1R treatments, as shown in Figure 2F.

### 2.2. Identification of DEGs in Different Treatments

**Figure 3:**
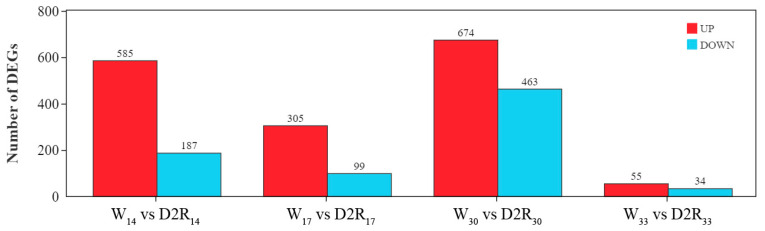
Number of differentially expressed genes (DEGs) in the drought–rewatering (D2R) treatment compared with the well-watered control (W). Data are from DEGs (with false discovery rate ˂ 0.05 and absolute value of log_2_(fold change) ≥ 1) at 14, 17, 30, and 33 days of the experiment (W_14_ vs. D2R_14_, W_17_ vs. D2R_17_, W_30_ vs. D2R_30_, and W_33_ vs. D2R_33_, respectively).

To understand the underlying mechanism regulating the growth-promoting effects of D2R treatment on tiller development in perennial ryegrass, RNA-seq analysis of the crowns of perennial ryegrass samples from the W and D2R treatments was performed at 14, 17, 30, and 33 d. A total of 2272 DEGs were obtained from the four comparison groups (W_14_ vs. D2R_14_, W_17_ vs. D2R_17_, W_30_ vs. D2R_30_, and W_33_ vs. D2R_33_) (Figure 3 and Table S1). The W_33_ and D2R_33_ comparison group had the lowest number of DEGs, with 55 upregulated genes and 34 downregulated genes in D2R_33_ compared with W_33_ (Figure 3). The W_30_ and D2R_30_ comparison group had the highest number of DEGs, including 674 upregulated genes and 463 downregulated genes in D2R_30_ compared with W_30_. The W_14_ and D2R_14_ comparison group had 772 DEGs with 585 upregulated genes and 187 downregulated genes in D2R_14_ compared with W_14_. The W_17_ and D2R_17_ comparison group had 404 DEGs with 305 upregulated genes and 99 downregulated genes in D2R_17_ compared with W_17_. In all four comparisons, the number of upregulated DEGs was more than that of downregulated DEGs (Figure 3).

### 2.3. WGCNA to Identify DEGs Associated with Tiller Number

**Figure 4:**
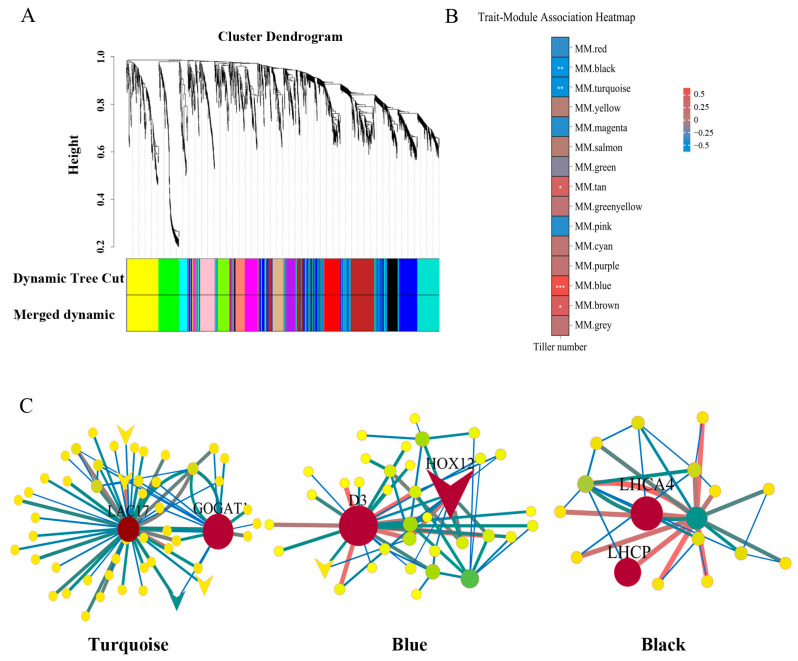
A global transcriptome and gene co-expression network related to tiller number in perennial ryegrass under well-watered control (W) and two cycles of drought–rewatering (D2R) treatments. (**A**) Cluster dendrogram indicating 15 clusters of differentially expressed genes (DEGs), with each cluster color-coded as a specific module. (**B**) Association analysis between tiller numbers and modules, * indicates significant association at *p* < 0.05, ** indicates significant association at *p* < 0.01, and *** indicates significant association at *p* < 0.001. Module membership (MM) as indicated by the color gradient represents the correlation between tiller numbers and modules, with red and blue values indicating positive and negative correlations, respectively. (**C**) The correlation networks of hub genes in each module. Hub genes identified by the highest weights in the weighted gene co-expression network analysis (WGCNA) within each network are highlighted in red and labeled based on their annotations.

WGCNA was employed to determine the regulatory networks and the specific genes that are associated with the effects of drought–rewatering cycles on promoting tillering development in perennial ryegrass. Based on the clustering among genes, 2272 DEGs were divided into 15 color-coded gene modules (Figure 4A) with varying degrees of association with tiller number (Figure 4B). Correlation coefficients between modules and tiller number showed that the blue, turquoise, black, tan, and brown modules had significant correlations with tiller number (Figure 4B, Table S2). By calculating the correlation significance between gene expression level and tiller number in each module, we found that the top three modules with the highest gene significance values were turquoise, blue, and black (Figure S1), which were further analyzed.

Hub genes were identified in the turquoise, blue, and black modules based on high module membership (MM > 0.7), high gene significance (GS > 0.9), and high kWithin (intramodular connectivity) (Figure 4C and Table S3). *Putative laccase-17* (*LAC17*) and *glutamate synthase 1 [NADH]* (*GOGAT1*) had the highest kWithin in the turquoise module (Figure 4C and Table 1). In addition, 12 transcription factors were identified in the turquoise module: five MYB transcription factors, two WRKY transcription factors, one bHLH transcription factor, one NAC transcription factor, one TCP transcription factor, one SRS transcription factor, and one Nin-like transcription factor (Table S3). *Homeobox-leucine zipper protein HOX12-like* (*HOX12*) and *F-box/LRR-repeat MAX2* homolog (*D3*) were in the 10 highest kWithin value genes and selected as candidate hub genes in the blue module (Figure 4C and Table 1). In the blue module, 11 transcription factors were identified: two NAC, two CO-like, one HD-ZIP, one ERF, one bHLH, one MYB_related, one C3H, one C2H2, and one FAR1 (Table S3). Based on the 10 highest kWithin value genes, *chlorophyll a-b binding protein/LHCII type I CAB* (*LHCP*) and *chlorophyll a-b binding protein 4* (*LHCA4*) were identified as candidate hub genes in the black module (Figure 4C and Table 1). There was no transcription factor found in the black module (Table S3).

### 2.4. Photosynthesis Played Significant Roles in Regulating Tiller Development under Two Cycles of Drought–Rewatering Treatment

**Figure 5:**
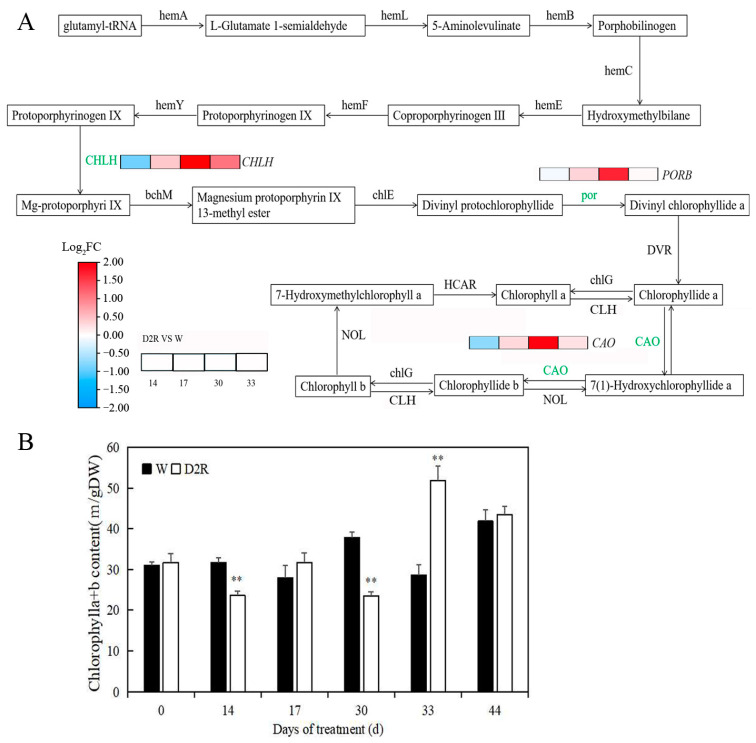
Chlorophyll metabolic pathway and chlorophyll content of well-watered control (W) and two cycles of drought–rewatering (D2R) treatments. (**A**) The diagram of chlorophyll metabolic. The normalized gene expression level at 14, 17, 30, and 33 days of the experiment is shown in a panel of blocks with colors from left to right. Color gradients from red to blue represent the log_2_FC value of the genes from upregulation to downregulation compared to W, respectively. (**B**) The chlorophyll concentrations of perennial ryegrass leaves in dry weight (DW). Vertical bars indicate the standard error of each treatment across six replications. ** indicates significant differences at *p* < 0.01. hemA, glutamyl-tRNA reductase; hemL, glutamate-1-semialdehyde 2,1-aminomutase; hemB, porphobilinogen synthase; hemE, uroporphyrinogen decarboxylase; hemF, coproporphyrinogen III oxidase; hemY, protoporphyrinogen/coproporphyrinogen III oxidase; CHLH, magnesium chelatase subunit H; bchM, magnesium-protoporphyrin O-methyltransferase; chlE, magnesium-protoporphyrin IX monomethyl ester (oxidative) cyclase; PORB, protochlorophyllide reductase B; DVR, divinyl chlorophyllide-a 8-vinyl-reductase; chlG, chlorophyll/bacteriochlorophyll a synthase; CLH, chlorophyllase; HCAR, 7-hydroxymethyl chlorophyll a reductase; NOL, chlorophyll (ide) b reductase; CAO, chlorophyllide a oxygenase.

The WGCNA analysis indicated that the black module was highly correlated with tiller development; in this module, photosynthesis (q-value, 2.84 × 10^−13^) and photosynthesis-antenna protein (q-value, 2.72 × 10^−30^) pathways were enriched (Figure S2 and Table S4). Chlorophyll is the primary pigment to capture light energy during photosynthesis. Transcriptome analysis indicated that the expression level of genes involved in chlorophyll synthetic pathways [*magnesium chelatase subunit H* (*CHLH*), *protochlorophyllide reductase B* (*PORB*), and *chlorophyllide a oxygenase* (*CAO*)] were all significantly upregulated at 30 d of the experiment in D2R compared to W control (Figure 5A). The contents of chlorophyll at different time points in the D2R and W treatments were then evaluated (Figure 5B). The results indicated that the chlorophyll content of the D2R treatment was significantly decreased at two drought periods at 14 and 30 days of the experiment compared to the W control and recovered to the control level at 17 days (the end of the first rewatering period). Interestingly, the chlorophyll content of D2R at 33 days (the second rewatering) was significantly higher than that of W (Figure 5B).

**Figure 6:**
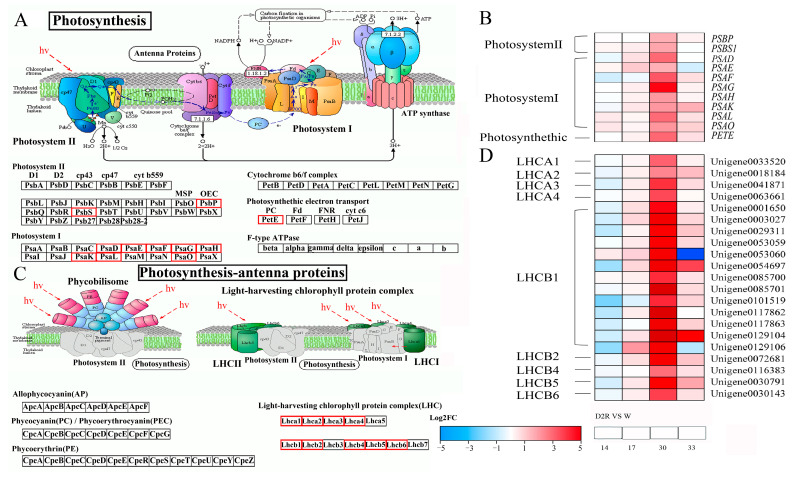
Differentially expressed genes (DEGs) related to the photosynthesis and photosynthesis-antenna protein pathways under well-watered control (W) and two cycles of drought–rewatering (D2R) treatments. (**A**) The Kyoto Encyclopedia of Genes and Genomes (KEGG) pathway of photosynthesis. (**B**) The expression of genes related to photosynthesis. (**C**) The KEGG pathway of photosynthesis-antenna proteins. (**D**) The expression of genes related to photosynthesis-antennaa proteins. Illustrations in A and C were obtained from the KEGG database. PsbA-Psb27, photosystem II structure proteins; PsaA-PsaX, photosystem I structure proteins; PetB-PetG, cytochrome b6/f complex proteins; Lhca1-Lhca5, light-harvesting complex I chlorophyll a/b binding protein; Lhcb1-Lhcb7, light-harvesting complex II chlorophyll a/b binding protein 1. Genes with significant changes in expression are indicated by red boxes. Color gradients from red to blue represent the log_2_FC of the genes in D2R from upregulation to downregulation compared to W, respectively.

To determine the response of photosynthetic genes in the crown tissue under D2R treatment, the expression of genes encoding proteins involved in the photosynthetic process [photosystem I (PSI), light-harvesting complex I (LHCI), and photosystem II (PSII), light-harvesting complex II (LHCII)] was summarized (Figure 6). There were 11 DEGs related to KEGG photosynthesis pathways, with eight genes related to PSI (*PSAD*, *PSAE*, *PSAF*, *PSAG*, *PSAH*, *PSAK*, *PSAL*, and *PSAO*), two related to PSII (*PSBP* and *PSBS1*), and one related to photosynthetic electron transport (*PETE*) (Figure 6A,B). Twenty-one DEGs related to photosynthesis-antenna proteins were identified, with four genes related to LHCI and 17 related to LHCII (Figure 6C,D). Furthermore, all 32 DEGs associated with photosynthesis and photosynthesis-antenna protein pathways were significantly upregulated at 30 d in the D2R compared to the control (Figure 6B,D). During rewatering at 33 d, the expression levels of most of those 32 genes were similar between the D2R and W (Figure 6B,D).

### 2.5. Validation of RNA-Seq Expression Data by qRT-PCR

**Figure 7:**
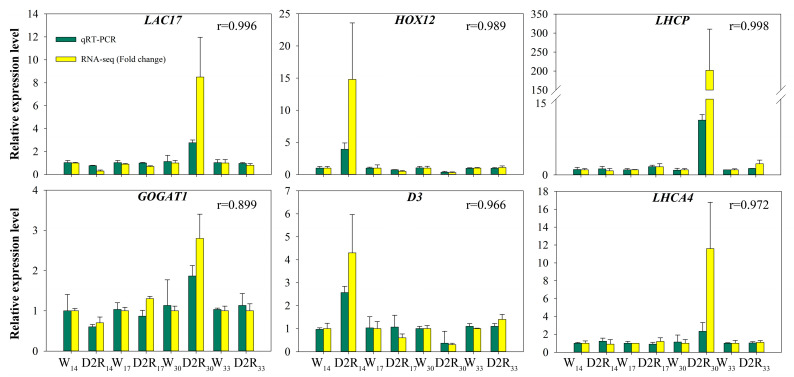
The relative gene expression analysis of six genes by qRT-PCR (green) and RNA-seq (yellow). The Pfaffl method was used to calculate the relative expression levels from qRT-PCR results using the W control as the baseline. The fold change values of RNA-seq were the average ratios of the FPKM for D2R group to the FPKM for W control. The Pearson’s correlation coefficients (r) are labeled in the upper right corner. Vertical bars indicate the standard error of each treatment across six replications.

To verify the RNA-seq results, six candidate hub genes were selected for a quantitative-reverse transcription polymerase chain reaction (qRT-PCR): *LAC17* and *GOGAT1* from the turquoise module, *D3* and *HOX12* from the blue module, as well as *LHCP* and *LHCA4* from the black module. High Pearson’s correlation coefficients (r) between the qRT-PCR and transcription data were identified, ranging from 0.899 to 0.998 (Figure 7), indicating the reliability of the transcriptomic results.

## 3. Discussion

### 3.1. Moderate Drought Conditions and Rewatering Cycles Could Lead to More Vigorous Growth

Plants in nature are confronted with the changing climate and various environmental stresses. Drought stress has been the major challenge for many crops and ecosystems globally [6]. Understanding the mechanism of how plants can respond to drought and adapt to adverse environmental conditions is an ongoing effort for crop improvement as well as the mitigation of climate change. Research reports have indicated that drought stress inhibits tiller formation and growth in plants [33,34]. In our study, we demonstrated that modifying watering schedule to introduce some drought stress can improve the growth of perennial ryegrass.

The tiller number in plants subjected to D2R treatment was equivalent to that in W at 30 days of the experiment (second drought stress), or higher than that in W after the second rewatering treatment at 33 and 44 d. The D2R treatment introduced relatively mild drought stress, as LRWC decreased to 78.8% and 78.9% at D2R_14_ and D2R_30_, respectively, and plants were able to recover quickly after rewatering. Plants subjected to D2R treatment also accumulated higher fresh and dry biomass (Figure 2). By contrast, plants subjected to longer period of drought from the D1R treatment, which caused more serve dehydration stress (LRWC was 59.1%), had a lower tiller number and biomass than those in W (Figure 2). Similar results were observed in *Leymus chinensis*; plants that experienced previous drought (SRWC between 35% and 50%) had higher biomass and tiller number after the second drought stress with 70 days of rewatering than the control plants [21]. Walter et al. observed an increase in photoprotective capacity and greater fresh biomass in *Arrhenatherum elatius* under two drought periods (about 60% and 70% LRWC for each time) than after a single drought (about 65% LRWC) [31]. However, some studies have shown the opposite results. Nosalewicz et al. [17] and Mastalerczuk and Borawska-Jarmułowicz [35] showed that both shoot and root biomass of *L. perenne* decreased with the increasing number of drought periods. Under more severe drought conditions, the capillary water capacity of the substrate was decreased from 70% to approximately 12%, and the LRWC was decreased from 80% to approximately 48%, and this can cause irreversible damage to plants [35]. This suggested that only plants that underwent mild to moderate drought conditions and rewatering (recovery) cycles could experience more vigorous growth. Combining the results from our study and others, it is necessary to rewater the plants when leaves show mild wilting symptoms to sustain normal plant growth or even promote tillering compared to those under well-watered conditions, whereas low LRWC (less than 70%) should be avoided.

### 3.2. Transcriptional Changes in Hub Genes Were Associated with Enhanced Tiller Development under Repeated Drought–Rewatering Treatment

A few transcriptome studies have been conducted to understand plant responses to drought–rewatering treatments [7,36,37]. Ding et al. [36] analyzed RNA-seq data from Arabidopsis leaves that were subjected to short periods of dehydration and rehydration (air-drying for 2 h and rehydration for 22 h) and compared them to pre-dehydration treatment. In contrast to the short cycles of dehydration–rewatering, we studied the plant responses to drought, simulating the actual conditions of perennial ryegrass grown in the field. RNA-seq data of the four time points comparing D2R and W treatments (W_14_ vs. D2R_14_, W_17_ vs. D2R_17_, W_30_ vs. D2R_30_, and W_33_ vs. D2R_33_) were obtained in our study. Moreover, we utilized crown tissues, which are valuable for research on the mechanisms of the effects of repeated drought and rewatering on tiller development in the Poaceae family.

**Figure 8:**
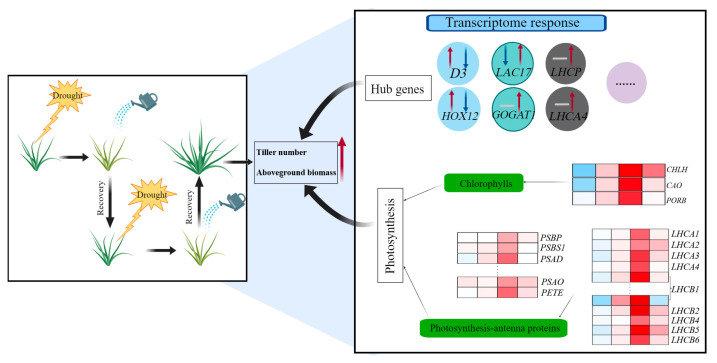
An overview of putative regulatory mechanisms of enhanced tiller development by two drought–rewatering cycles (D2R). The black box on the left describes the D2R treatment, which significantly increased the number of tillers and aboveground biomass at the conclusion of the experiment. The transcriptome response analyzed by weighted gene co-expression network analysis (WGCNA), combining transcriptome data and tiller numbers, are summarized in the black box on the right. The red arrow pointing up indicates a significant increase, the blue arrow pointing down indicates a significant decrease, and the gray horizontal line indicates no significant change. Red and blue colors in the heat map represent upregulation and downregulation compared to the well-watered control, respectively.

In addition, 2272 DEGs from the RNA-seq data were divided into 15 gene modules based on clustering analysis (Figure 4); a co-expression regulatory network based on WGCNA was established in this study (Figure 4). Hub genes, *D3*, *HOX12*, *LAC17*, *GOGAT1*, *LHCP*, and *LHCA4* were identified based on high MM, GS, and kWithin values (Figure 4 and Table 1); the transcriptome data were further validated by qRT-PCR (Figure 7). According to the changes in gene expression levels at different time points, the six hub genes were divided into three expression patterns: two genes (*D3*, *HOX12*) with significantly higher expression at D2R_14_ but lower expression at D2R_30_ compared to W control; one gene (*LAC17*) with significantly lower expression at D2R_14_ but higher expression at D2R_30_; and three genes (*GOGAT1*, *LHCP*, *LHCA4*) with no significant change at D2R_14_ but significantly higher expression at D2R_30_ (Figure 7 and Figure 8, Table S1).

In this study, the hub genes *D3* and *HOX12* were found to be upregulated at D2R_14_ after the first drought stress but downregulated at D2R_30_, which was consistent with fewer tillers observed at D2R_14_ and more tillers at D2R_30_. Studies have shown that strigolactones (SLs) are produced by plant roots and transported upward to axillary buds; these play a key role in inhibiting the elongation and growth of axillary buds [38]. *D3/MAX2* was involved in SL signaling, which inhibits tiller development; an increased tiller number was observed in a *d3/max2* mutant [39,40]. In another study, Bu et al. demonstrated that *max2* mutant was hypersensitive to drought stress compared with wild-type Arabidopsis (*A. thaliana*) [41]. Those results indicated that *MAX2* participated in drought stress response as well as in tiller development, making it a highly valuable candidate for explaining the promotion effect of tiller growth under repeated drought–rewatering treatment. Furthermore, Liu et al. described the mutual regulatory relationship between SL and ABA, indicating that the SL signal pathway can up-regulate the expression of *OsHOX12* and increase the expression of the key gene *OsNCED1* for ABA synthesis; therefore, *OsHOX12* can promote ABA synthesis at the base of the stem, which inhibits tillering [42].

After the second cycle of drought, *LAC17*, *GOGAT1*, *LHCP*, and *LHCA4* were upregulated at D2R_30_. Laccases are the largest component of the multi-copper oxidoreductases family [43], and laccases like *LAC17* are involved in lignin synthesis, degradation, and detoxification of lignin derivatives [44,45]. Research has shown that lignin biosynthesis can also positively affect the number of tillers [46,47]. *GOGAT1* is involved in glutamate biosynthesis, and the tiller number of single activation tagging mutant *OsGOGAT1* is lower than that of the wild type; however, double activation tagging mutant *OsAMT1;2/OsGOGAT1* had more tillers than the wild type in rice [48,49]. Higher *GOGAT* activity and gene expression have been reported to contribute to higher nitrogen translocation efficiency and nitrogen use efficiency, thus improving plant yield [50]. The findings by Liu et al. [50], together with enhanced photosynthesis indicated by the upregulation of photosynthesis-related genes and increase in chlorophyll content, could also explain the enhanced tiller numbers and aboveground biomass in this study. The high correlation between *GOGAT1* expression and tiller number under D2R treatment also suggested that *GOGAT1* is a candidate gene for studying tiller development (Figure 4C, Table 1). Pintó-Marijuan et al. found that significant modification of the photosystems’ antenna and reaction centers was observed in heartleaf iceplants (*Aptenia cordifolia*) that were subjected to previous water stress cycles, and this led to higher PSII efficiency than that in plants exposed to drought for the first time [51]. *LHCP* and *LHCA4* encode chlorophyll a-b binding protein/LHCII type I CAB and chlorophyll a-b binding protein 4, respectively. The expression of these two hub genes was consistent with the change in drought response at D2R_14_ and the significant increase in tiller number at D2R_30_. The upregulation of these hub genes in D2R_30_ suggested that two cycles of drought–rewatering promote the tiller development of perennial ryegrass by enhancing nutrient uptake efficiency and photosynthesis.

### 3.3. Enhanced Expression of Photosynthesis and Photosynthesis-Antenna Genes Contributed to Increased Tiller Growth of Perennial Ryegrass under Repeated Drought–Rewatering

Photosynthesis (q-value, 2.84 × 10^−13^) and photosynthesis-antenna protein pathways (q-value, 2.72 × 10^−30^) were among the highly enriched pathways in the black modules in this study, and these were chosen for further analysis (Figure S2). Photosynthesis is often used as an important indicator for evaluating plant health under abiotic stress conditions [52]. Under environmental stresses, the photosynthetic ability of plants usually declines sharply, leading to the inhibition of plant growth and development, such as reducing the production of new tillers [53,54]. However, the expression level of DEGs related to photosynthesis and photosynthesis-antenna protein pathways was significantly upregulated at D2R_30_ (second drought stress), which was the opposite of the expression pattern at D2R_14_ (first drought stress). Although the content of chlorophyll was not significantly different between W_30_ and D2R_30_, it was remarkably higher 3 d after the second rewatering at D2R_33_ than that in W_33_ (Figure 5B). This indicated that after undergoing the first drought stress, the photosynthetic capacity of plants under the second drought stress could be improved, thereby promoting the development of tillers. LHCI and LHCII are used as light-absorbing antenna systems in PSI and PSII, respectively, while the pigments in LHCI and LHCII are mainly chlorophyll a and chlorophyll b, respectively [52,55]. The KEGG and WGCNA analysis results revealed that three DEGs, including the synthetic pathway genes *CHLH*, *PORB*, and *CAO*, were enriched in chlorophyll metabolism. The results showed that the expression of *CHLH*, *POR*, and *CAO* was significantly upregulated at D2R_30_ (Figure 5). This may explain the higher chlorophyll content at D2R_33_. In the co-expression network of the three modules, 32 genes related to PSI/II and LHC were identified (Figure 6). Given the important role of LHC in light energy capture, the downregulated expression of LHC-encoding genes may decrease light utilization efficiency [52]. These results showed that all 32 DEGs of the photosynthesis pathway were significantly upregulated at D2R_30_. According to our WGCNA analysis, light-harvesting chlorophyll a/b binding proteins *LHCP* and *LHCA4* were the hub genes in the co-expression network (Figure 4C, Table 1). Although many studies have discussed the role of photosynthesis and photosynthesis-antenna proteins in drought–rewatering [21,56,57], there are also studies highlighting the important roles of PSI/II-related and LHC-encoding genes on plant development (such as plant height, biomass, etc.) [58,59], but few studies have illustrated the role of PSI/II-related and LHC-encoding genes in plant tiller development. Our study focused on correlating tiller number and transcriptome data under D2R and W treatment through WGCNA and identified the pathways and genes related to photosynthesis. Hence, PSI/II-related and LHC-encoding genes were demonstrated to be of great significance for the maintenance of photosynthesis and the promotion of tiller development under D2R treatment in perennial ryegrass.

This study revealed a dynamic transcriptional landscape of D2R treatment response in perennial ryegrass and enhanced our knowledge of how plants respond to drought stress. As shown in Figure 8, repeated drought–rewatering treatment increased the tiller number and aboveground biomass of perennial ryegrass. Through the analysis of the transcriptome response of crown tissue, enhanced photosynthesis capacity as well as change in the transcriptional level of six hub genes were associated with enhanced tiller development under repeated drought–rewatering treatment compared to well-watered control.

## 4. Material and Methods

### 4.1. Plant Material and Growth Conditions

Fifteen 10-day-old seedlings of perennial ryegrass (cv. ‘Monterey Ⅲ’) were each planted in a polyvinyl chloride tube (20 cm height, 10 cm diameter) filled with fritted clay in a greenhouse at the College of Agro-grassland Science, Nanjing Agricultural University, Nanjing, China. Treatments were carried out in the growth chamber (MT8070iE, Xubang, Zhengzhou, China) with plant-growth light (LU400W/PSL/T/E40, GE Lighting, East Cleveland, OH, USA). The growth conditions were set as follows: day/night temperatures at 25/15 °C and 70% relative humidity with 14 h photoperiod of 500 μmol m^−2^ s^−1^ photosynthetically active radiation at the canopy level. The tubes were randomly relocated in the chambers every other day to avoid spatial environmental variations in the growth chamber. Plants were fertilized weekly with half-strength Hoagland’s nutrient solution before treatment initiation and during the rewatering period after 30 d for all treatments [60]. One additional fertilization with half-strength Hoagland’s nutrient solution was applied at 14 d for treatment W and D2R. Nutrient deficiency was not observed during the study.

### 4.2. Treatments and Experimental Design

After 7 d acclimation in the growth chamber, the plants were subjected to three different irrigation treatments (shown in Figure 1). In the well-watered control treatment (W), daily irrigation was applied to maintain the soil relative water content (SRWC) at full capacity (86.5–89.4%) throughout the experiment (44 d). In the D2R treatment, the SRWC was gradually reduced by withholding water until the leaves started to wilt at 14 d (D2R_14_, the subscript number denotes the day of the experiment), then re-watered for three days (D2R_14_ to D2R_17_). Drought stress was induced again until the plant leaves began to wilt at 30 d (D2R_30_). After that, the plants were re-watered, and the SRWC was maintained at the control level for 14 d (D2R_30_ to D2R_44_). In the D1R treatment, water was withheld for 30 d (D1R_30_) creating relatively severe drought stress and then re-watered for 14 d (D1R_30_ to D1R_44_). Photos of plant performance under different treatments were taken at 44 d. Tiller buds longer than 1 cm were designated as a tiller. The number of tillers were counted at each time point after treatment. Crowns at 14, 17, 30, and 33 d were harvested and frozen immediately in liquid nitrogen and stored at −80 °C for W and D2R treatments. Samples for 14 and 30 d were collected at the end of the first and second drought stress, respectively, but before the first and second rewatering. At 44 d, the fresh weight (FW) of the aboveground tissue was determined; then, the aboveground tissue for each sample was dried at 80 °C in an oven for three days to determine dry weight (DW). Due to the destructive sampling of crowns, a total of 90 tubes were prepared with 30 tubes for each treatment. At a given time point, each tube served as a biological replicate, and each treatment had six replicates. Furthermore, to ensure the accuracy of the experiment, three independent runs of experiments were carried out, and similar results were obtained.

### 4.3. Determination of Soil Relative Water Content and Leaf Relative Water Content

Soil relative water content (SRWC) was recorded as the gravimetric water content in each pot using the equation as reported by Wang et al. [61]: SRWC = (actual tube weight − tube weight with dried soil) × 100%/(tube weight with saturated soil − tube weight with dried soil). Each day, all the tubes were weighed in W treatment during the experiment. For D2R and D1R treatment, tubes were weighed during rewatering stages to maintain SRWC at full capacity.

To measure leaf relative water content (LRWC), fully expanded leaves were first weighed (fresh weight, FW) and then soaked in distilled water for 24 h to obtain the turgid weight (TW). After an initial incubation at 105 °C in the oven for 30 min, dry weight (DW) was recorded after leaf segments were dried at 80 °C in an oven for 48 h. The LRWC was calculated as LRWC (%) = (FW − DW)/(TW − DW) × 100.

### 4.4. Measurements of Chlorophyll Content

Extractions and measurements of chlorophyll were carried out using a previously described method by Tait and Hik [62]. About 0.1 g of mature fresh leaves were immersed in 8 mL of dimethyl sulfoxide (DMSO) for 72 h in the dark to extract chlorophyll. The absorbance of the supernatants was measured at 665 and 649 nm using spectrophotometry (Spectronic in Instruments, Rochester, NY, USA). Chlorophyll content was calculated following the formula: Chl content (mg g^−1^ DW) = [(12.19 × A665 − 3.45 × A649 + 21.99 × A649 − 5.32 × A665) × 0.01]/dry weight [62].

### 4.5. Total RNA Isolation, Library Preparation, and Transcriptome Sequencing

Crowns from plants that were subjected to W and D2R treatments were used for transcriptome analysis. Each treatment had three replicates. Total RNA was extracted using Trizol reagent kit (Invitrogen, Carlsbad, CA, USA) according to the manufacturer’s protocol. RNA quality was assessed on an Agilent 2100 Bioanalyzer (Agilent Technologies, Palo Alto, CA, USA) as well as via agarose gel electrophoresis. RNA-seq was performed using Illumina novaseq 6000 by Gene Denovo Biotechnology Co. (Guangzhou, China).

### 4.6. Differential Expressed Genes (DEGs), Weighted Gene Co-Expression Network Analysis (WGCNA), Gene Ontology (GO), and Pathway Enrichment Analysis

Differential expression analysis between D2R and W treatments was performed using DESeq2 (v1.25.9) [63]. The genes/transcripts with a false discovery rate (FDR) ˂ 0.05 and the absolute value of log_2_FC (fold change) ≥ 1 were considered differentially expressed genes/transcripts.

DEGs were analyzed by using the WGCNA package in R (v3.4.1) to construct co-expression networks [64]. The networks were visualized using Cytoscape 3.3.0 [65].

Gene Ontology (GO) enrichment analysis identified GO terms that are significantly enriched in the DEGs compared to the genome background [66]. The Kyoto Encyclopedia of Genes and Genomes (KEGG) pathway enrichment analysis identified significantly enriched metabolic pathways or signal transduction pathways in DEGs compared to the whole genome background [67]. GO terms and KEGG pathways were considered significantly enriched in DEGs with FDR ≤ 0.05.

### 4.7. Validation and Expression Analysis by qRT-PCR

The first strand cDNA synthesis was carried out using MonScript^TM^ RTIII Super Mix with dsDNase (Two-Step) kit (Monad Biotech, Wuhan, China) according to the manufacture’s protocol. The cDNA samples were diluted 25× in DNase-free water prior to quantitative PCR (qPCR) analysis. All the primers used are listed in Supplementary Table S5. Dye-based relative qRT-PCR with SYBR^®^ Green was adopted to measure cDNA amplification. The qRT-PCR assays were performed using Roche LightCycler^®^480II (Roche Diagnostic, Rotkreuz, Switzerland) with MonAmp^TM^ ChemoHS qPCR Mix (Monad Biotech) to detect gene expression levels. The PCR condition was set as initially denatured for 10 min at 95 °C followed by 40 cycles of PCR (95 °C for 15 s, 60 °C for 15 s, and 72 °C for 20 s). Data were collected at 65 °C in each cycle. *LpeIF4A* (*eukaryotic initiation factor 4 alpha*) was used as the reference gene for perennial ryegrass [68]. The qPCR amplification efficiency was determined for each primer pair based on calibration curves with slopes and y-intercepts [69]. The relative transcript levels of the target genes were determined using the equations described by Pfaffl [70]. All transcript levels were expressed as the mean ± standard error (SE) of the six biological replicates.

### 4.8. Statistical Analysis

The software SPSS Version.19 (SPSS Inc., Chicago, IL, USA) was used to perform one-way variance (ANOVA) analyses. The statistical significance of data was assessed utilizing the least significant difference (LSD) test at *p* < 0.05. Photos for plants were taken using a single-lens reflex camera (Nikon D5100, Bangkok, Thailand).

## 5. Conclusions

Plants under two cycles of drought–rewatering treatment showed an 40.9% increase in tiller number and a 28.3% increase in aboveground biomass compared to the well-watered control, suggesting that irrigation practices can be modified to reduce water usage but also improve plant growth. WGCNA analysis correlating transcriptome data with tiller number suggested that genes related to photosynthesis and photosynthesis antenna proteins were differentially expressed to promote tiller development under D2R treatment. Additionally, SL signaling, glutamate biosynthesis, chlorophyll-a-b-related genes, and HD-zip transcription factors are candidates for the future investigation of tiller development and desirable traits for drought tolerance (Figure 8). Future study is also needed to test these irrigation strategies under field conditions. In addition, whether the upregulation of genes involved in improving nitrogen nutrient uptake efficiency (as the expression of *GOGAT* was accumulated at D2R_30_) leads to enhanced photosynthesis capability needs to be further investigated in future research. Moreover, functional analyses of the hub genes identified in this study are essential to understanding the molecular mechanisms of these genes in the future.

## Figures and Tables

**Table 1:** Candidate hub genes of the three modules.

Gene ID.	Gene Name	Annotation of Unigenes	kWithin
Turquoise			
Unigene0061484	*LAC17*	XP_051186142.1 putative laccase-17 [*Lolium perenne*]	74.3
Unigene0109552	*GOGAT1*	XP_003566997.1 glutamate synthase 1 [NADH], chloroplastic [*Brachypodium distachyon*]	70.8
Blue			
Unigene0122450	*HOX12*	XP_047088374.1 homeobox-leucine zipper protein HOX12-like [*Lolium rigidum*]	63.3
Unigene0047820	*D3*	XP_003564315.1 F-box/LRR-repeat MAX2 homolog [*Brachypodium distachyon*]	58.8
Black			
Unigene0072681	*LHCP*	KAE8804416.1 Chlorophyll a-b binding protein, chloroplastic [*Hordeum vulgare*]	36.8
Unigene0063661	*LHCA4*	XP_010235157.1 chlorophyll a-b binding protein 4, chloroplastic [*Brachypodium distachyon*]	36.2

kWithin: The gene connectivity within a module.

## Data Availability

The RNA-seq raw data were deposited into the National Center for Biotechnology Information (NCBI) Gene Expression Omnibus database (accession number: PRJNA917112).

## Supplementary Materials

The following supporting information can be downloaded at https://www.mdpi.com/article/10.3390/plants13060854/s1, Figure S1: The mean gene significance between module and tiller number. Error bars indicate standard errors. Figure S2: The Gene Ontology (GO) and the Kyoto Encyclopedia of Genes and Genomes (KEGG) enrichment and correlation network of major genes from the turquoise, blue, and black modules. (A) The GO enrichment in the modules. (B) The KEGG enrichment in the modules. Numbers next to the horizontal bars indicate the number of genes in each category and numbers in the parentheses represent –log_2_(Qvalue). Table S1: The expression in four comparison groups genes. Table S2: Information of the five modules closely related to tiller number. Table S3: Gene connectivity in three modules. Table S4: Top10 pathway of three modules. Table S5: Primers and related information list used in qRT-PCR.
