# Supplementary figures and images for: Transcriptome-Based Weighted Gene Co-Expression Network Analysis Reveals the Photosynthesis Pathway and Hub Genes Involved in Promoting Tiller Growth under Repeated Drought–Rewatering Cycles in Perennial Ryegrass
Source: Plants (Basel). 2024 Mar 15;13(6):854. doi: 10.3390/plants13060854 (PMC10976046; doi:10.3390/plants13060854)

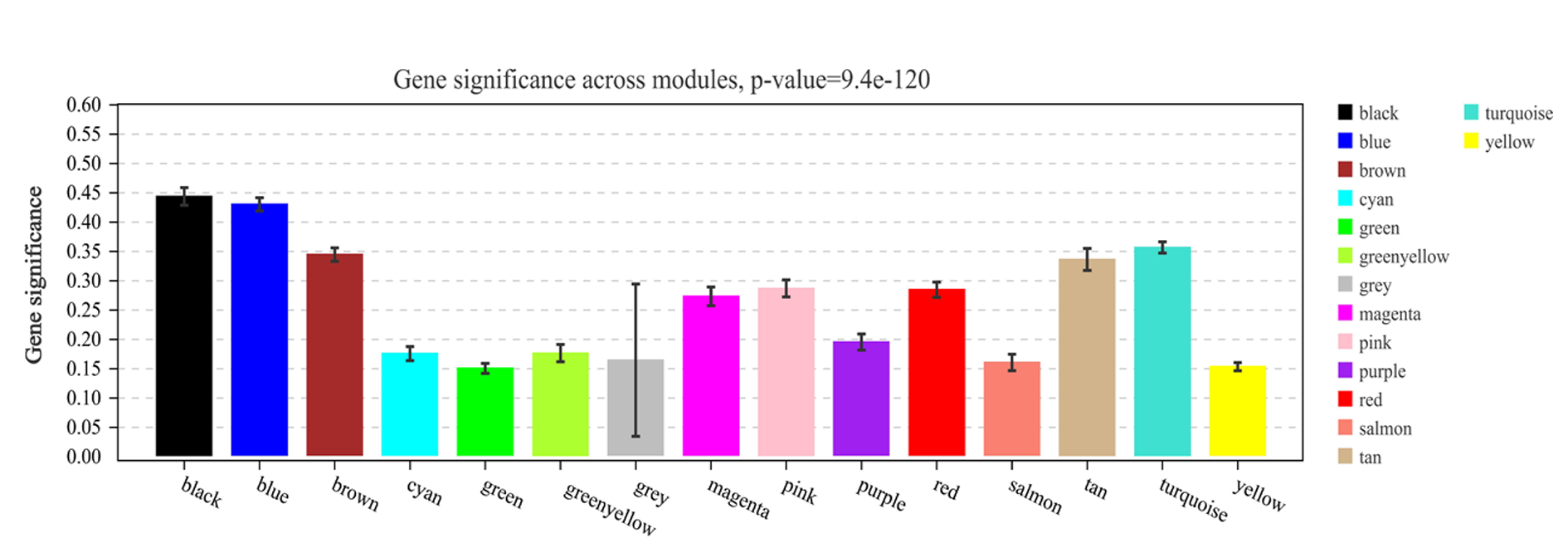

Supplement: Supplementary file 1 [file plants-13-00854-s001.zip › Fig.S1.tif]

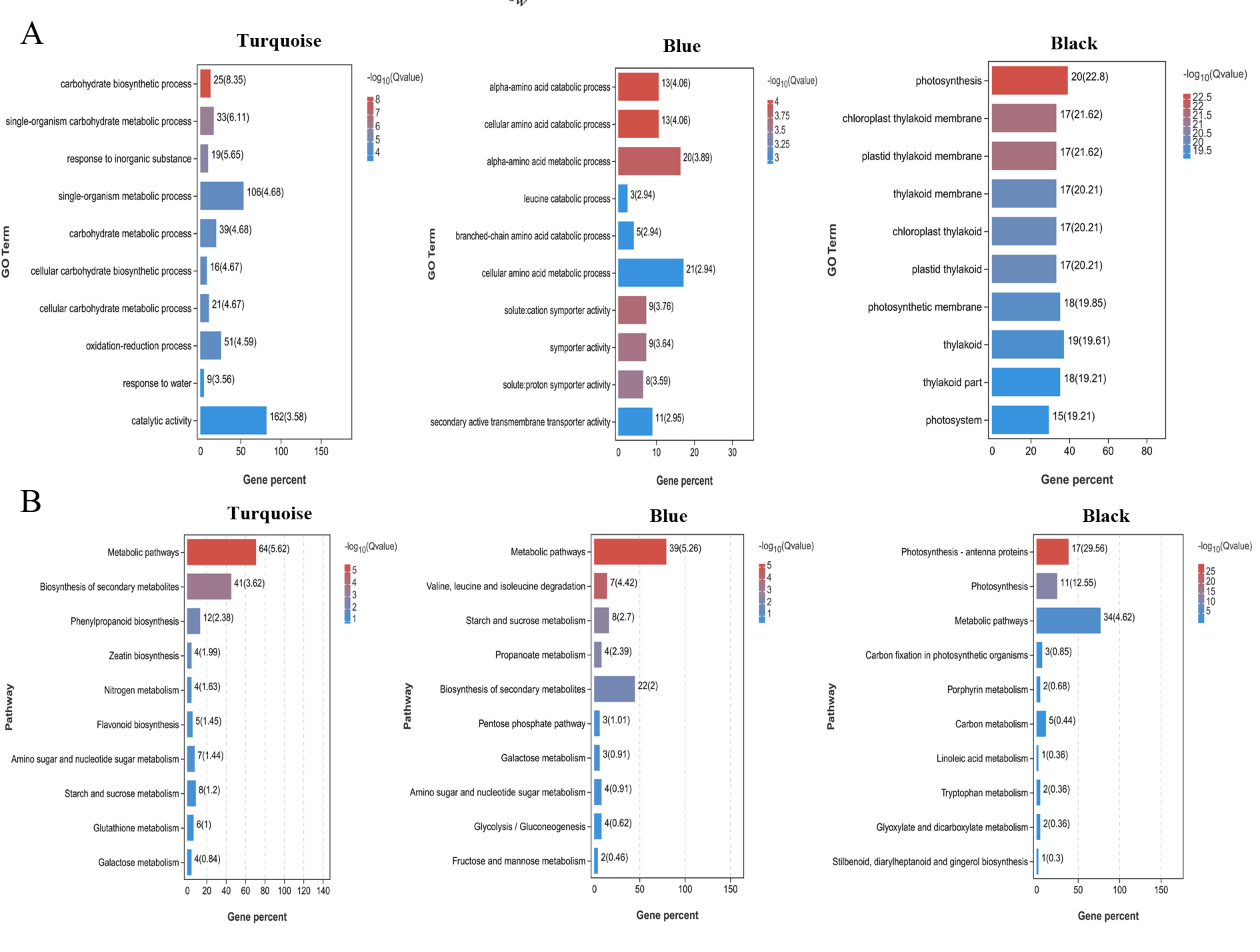

Supplement: Supplementary file 1 [file plants-13-00854-s001.zip › Fig.S2.tif]
